# Phenological mismatch in Arctic‐breeding shorebirds: Impact of snowmelt and unpredictable weather conditions on food availability and chick growth

**DOI:** 10.1002/ece3.5248

**Published:** 2019-05-16

**Authors:** Sarah T. Saalfeld, Daniel C. McEwen, Dylan C. Kesler, Malcolm G. Butler, Jenny A. Cunningham, Andrew C. Doll, Willow B. English, Danielle E. Gerik, Kirsten Grond, Patrick Herzog, Brooke L. Hill, Benjamin J. Lagassé, Richard B. Lanctot

**Affiliations:** ^1^ Migratory Bird Management Division U.S. Fish and Wildlife Service Anchorage Alaska; ^2^ Limnopro Aquatic Science, Inc. South Haven Minnesota; ^3^ The Institute for Bird Populations Point Reyes Station California; ^4^ Department of Biological Sciences North Dakota State University Fargo North Dakota; ^5^ Department of Fisheries and Wildlife Sciences University of Missouri Columbia Missouri; ^6^ Denver Museum of Nature & Science Denver Colorado; ^7^ National Wildlife Research Centre Carleton University Ottawa Ontario Canada; ^8^ College of Fisheries and Ocean Sciences University of Alaska Fairbanks Fairbanks Alaska; ^9^ Department of Molecular & Cell Biology University of Connecticut Storrs Connecticut; ^10^ Institut für Biologie, Zoologie - Molekulare Ökologie Martin-Luther-Universität Halle-Wittenberg Halle Germany; ^11^ Department of Biology and Wildlife University of Alaska Fairbanks Fairbanks Alaska; ^12^ Department of Integrative Biology University of Colorado Denver Denver Colorado

**Keywords:** Arctic, chick growth rates, insect emergence, invertebrate availability, phenological mismatch, shorebirds, trophic mismatch

## Abstract

The ecological consequences of climate change have been recognized in numerous species, with perhaps phenology being the most well‐documented change. Phenological changes may have negative consequences when organisms within different trophic levels respond to environmental changes at different rates, potentially leading to phenological mismatches between predators and their prey. This may be especially apparent in the Arctic, which has been affected more by climate change than other regions, resulting in earlier, warmer, and longer summers. During a 7‐year study near Utqiaġvik (formerly Barrow), Alaska, we estimated phenological mismatch in relation to food availability and chick growth in a community of Arctic‐breeding shorebirds experiencing advancement of environmental conditions (i.e., snowmelt). Our results indicate that Arctic‐breeding shorebirds have experienced increased phenological mismatch with earlier snowmelt conditions. However, the degree of phenological mismatch was not a good predictor of food availability, as weather conditions after snowmelt made invertebrate availability highly unpredictable. As a result, the food available to shorebird chicks that were 2–10 days old was highly variable among years (ranging from 6.2 to 28.8 mg trap^−1^ day^−1^ among years in eight species), and was often inadequate for average growth (only 20%–54% of Dunlin and Pectoral Sandpiper broods on average had adequate food across a 4‐year period). Although weather conditions vary among years, shorebirds that nested earlier in relation to snowmelt generally had more food available during brood rearing, and thus, greater chick growth rates. Despite the strong selective pressure to nest early, advancement of nesting is likely limited by the amount of plasticity in the start and progression of migration. Therefore, long‐term climatic changes resulting in earlier snowmelt have the potential to greatly affect shorebird populations, especially if shorebirds are unable to advance nest initiation sufficiently to keep pace with seasonal advancement of their invertebrate prey.

## 1 INTRODUCTION

The ecological consequences of climate change have been recognized in numerous species, with documented changes occurring to morphology (Gardner, Peters, Kearney, Joseph, & Heinsohn, 2011; Millien et al., 2006; Sheridan & Bickford, 2011; Teplitsky & Millien, 2014; van Gils et al., 2016), distributions (Austin & Rehfisch, 2005; Parmesan & Yohe, 2003; Thomas & Lennon, 1999), and phenology (Crick, Dudley, Glue, & Thomson, 1997; Forchhammer, Post, & Stenseth, 1998; Hovel, Carlson, & Quinn, 2017; McDermott & DeGroote, 2017; Parmesan & Yohe, 2003; Post, Forchhammer, Stenseth, & Callaghan, 2001; Stenseth et al., 2002; Walther et al., 2002). By adjusting phenology, individuals can time life‐history events so that peak food demands of developing young coincide with peak prey availability (Bronson, 1985; Durant, Hjermann, Ottersen, & Stenseth, 2007; Visser, Holleman, & Gienapp, 2006). However, organisms within different trophic levels may respond to changes in their environment at different rates (Cohen, Lajeunesse, & Rohr, 2018; Thackeray et al., 2016), potentially resulting in phenological mismatches between predators and their prey (Both, Asch, Bijlsma, Burg, & Visser, 2009; Brook, Leafloor, Abraham, & Douglas, 2015; Doiron, Gauthier, & Lévesque, 2015; Durant et al., 2007; Gaston, Gilchrist, Mallory, & Smith, 2009; Harrington, Woiwod, & Sparks, 1999; Visser et al., 2006; Visser, Noordwijk, Tinbergen, & Lessells, 1998).

Phenological mismatch may be especially important in the Arctic (Bart & Johnston, 2012), which has been affected more by climate change than other regions, resulting in earlier, warmer, and longer summers (Callaghan et al., 2005; Hodgkins, 2014; Serreze & Francis, 2006). Shorebirds comprise a large portion of the avian fauna breeding in the Arctic and are an ideal taxon to investigate phenological mismatch. These tundra‐nesting insectivores time their long‐distance migrations using a combination of endogenous and photoperiod cues (Karagicheva et al., 2016; Piersma, Brugge, Spaans, & Battley, 2008), but rely on a short pulse of abundant food whose seasonal emergence is dictated by local climatic conditions on the breeding grounds (Bolduc et al., 2013; Danks, 1999; Tulp & Schekkerman, 2008). In fact, several studies have shown that both Subarctic‐ and Arctic‐breeding shorebirds (Gill et al., 2014; Grabowski, Doyle, Reid, Mossop, & Talarico, 2013; Liebezeit, Gurney, Budde, Zack, & Ward, 2014; Saalfeld & Lanctot, 2017) and their invertebrate prey (Braegelman, 2016; Tulp & Schekkerman, 2008) have advanced their phenologies with recent climate change. Shorebird advancement rates, however, have not kept pace with advancing conditions (Grabowski et al., 2013; Saalfeld & Lanctot, 2017). Thus, there now appears to be several instances of phenological mismatch between the timing of shorebird hatch and peak invertebrate availability, although variability exists among species and sites (Kwon et al., 2019; McKinnon, Picotin, Bolduc, Juillet, & Bêty, 2012; Reneerkens et al., 2016; Senner, Stager, & Sandercock, 2017).

Past studies on phenological mismatch in Arctic‐breeding shorebirds have often focused on the timing of insect emergence as it relates to the date of shorebird egg hatching when defining phenological mismatch. However, this approach does not account for the amount of food needed for adequate growth and survival of young, and thus, may not be directly related to an individual's fitness (Green, Greenwood, & Lloyd, 1977; Tulp & Schekkerman, 2008). Indeed, several studies have shown that shorebird chick growth and survival rates are predominately influenced by invertebrate availability—not simply timing of hatch relative to peak insect emergence (McKinnon, Nol, & Juillet, 2013; Pearce‐Higgins & Yalden, 2002, 2004; Reneerkens et al., 2016; Schekkerman, Tulp, Piersma, & Visser, 2003; Senner et al., 2017). While it is often assumed that hatching shortly prior to peak insect emergence will result in greatest food availability for chicks, this may not always be the case. For example, invertebrate availability in the Arctic depends not only on the timing and magnitude of insect (largely dipteran) emergence but also on daily invertebrate activity levels that affect the ability of shorebirds to detect their prey (Bolduc et al., 2013; Schekkerman, Roomen, & Underhill, 1998; Schekkerman et al., 2003; Tulp & Schekkerman, 2008). Both factors are influenced by weather—insect emergence is controlled by cumulative temperatures or temperature thresholds (Bolduc et al., 2013; Butler, 1980; Danks, 1999; Høye & Forchhammer, 2008; Tulp & Schekkerman, 2008), while invertebrate activity is controlled by daily conditions (e.g., temperature, wind, precipitation; Bolduc et al., 2013; Schekkerman et al., 1998; Schekkerman et al., 2003; Tulp & Schekkerman, 2008). Therefore, variability in seasonal weather patterns may cause fluctuations both in the timing of insect emergence and in prey activity patterns, resulting in complex and potentially quite variable patterns of food availability during the avian breeding season. As a result, even if chicks hatch during peak insect emergence, there is no guarantee they will be able to find sufficient food if invertebrate activity decreases thereafter. Thus, relying on the timing of peak insect emergence as it relates to the date of shorebird egg hatching when defining phenological mismatch ignores the fact that having a sufficient amount of food for adequate growth during development is likely more important for an individual's fitness than is timing of hatch in relation to peak insect emergence.

To address this shortcoming, we estimated phenological mismatch over a 7‐year period in relation to food availability and chick growth rates in a community of Arctic‐breeding shorebirds experiencing advancement of environmental conditions (i.e., snowmelt; Saalfeld & Lanctot, 2017). Specifically, we (a) describe the inter‐ and intra‐annual variation in available invertebrate biomass in relation to snowmelt and seasonal weather conditions, (b) estimate phenological mismatch between timing of peak insect emergence and shorebird hatch relative to timing of snowmelt, (c) determine how the degree of phenological mismatch relates to food availability and growth rates of chicks, and (d) determine how timing of hatch with respect to snowmelt influences food availability of chicks for eight shorebird species.

## 2 MATERIALS AND METHODS

### 2.1 Study area

From 2010 to 2016, we collected data on shorebird nesting, invertebrate availability, and environmental variables at six 36‐ha plots near Utqiaġvik (formerly Barrow), Alaska (see Saalfeld & Lanctot, 2017). Data on chick growth were collected in 2013–2016. We divided all plots into 144 quadrats (50 × 50 m) using wooden stakes placed every 50 m to facilitate data collection. Habitat within the study plots consisted mainly of tundra dominated by sedges, grasses, and moss interspersed with small ponds. Thus, plots were a mosaic of low, wet marsh habitat and higher, well‐drained upland habitat (Brown, Everett, Webber, MacLean, & Murray, 1980).

### 2.2 Data collection

#### 2.2.1 Timing of snowmelt

Timing of snowmelt affects shorebird nest initiation dates by controlling when suitable habitat and food resources become available (Grabowski et al., 2013; Green et al., 1977; Liebezeit et al., 2014; Meltofte, 1985; Meltofte, Høye, Schmidt, & Forchhammer, 2007; Saalfeld & Lanctot, 2017; Smith, Gilchrist, Forbes, Martin, & Allard, 2010). Therefore, we estimated the percentage of snow cover to the nearest 5% within thirty‐six 50 × 50 m quadrats (25% of the plot) equally spaced throughout each 36‐ha study plot every 2–5 days until ≤10% snow cover remained. We then determined the mean percent snow cover across all 36 quadrats for each plot on a given date, and linearly regressed these values through time to determine the date when 20% snow cover was present on each plot in each year. We chose 20%, as it could be calculated in almost all years and plots (see exception below) and <11% of nests were initiated prior to this date. While several studies have used 50% as their cutoff value (Grabowski et al., 2013; Smith et al., 2010; but see Liebezeit et al., 2014 that used 5%), our annual date for 20% snow cover was highly correlated (*r* = 0.91) with the date of 50% snow cover for 11 years when data were available (i.e., 2004–2014). Thus, the use of the 20% cutoff value likely had little impact on our results in comparison to other cutoff values. In 2016, snow was present, but covered <20% on one plot during the first snow survey. Therefore, because winter winds keep snow from accumulating on the tundra and snow melts rapidly once temperatures reach 0°C, we used the date prior to the first snow survey as a conservative estimate of 20% snow cover for this plot.

#### 2.2.2 Invertebrate availability

We used 10–16 modified “Malaise” pitfall traps equally distributed among mesic and xeric tundra habitats to capture available invertebrates throughout the nesting season. These traps consisted of a 38 × 5 × 7 cm plastic container placed at ground level that captured ambulatory invertebrates, and a 36 × 36 cm mesh screen placed perpendicular above the container to capture aerial invertebrates that hit the screen and fell into the trap (Brown et al., 2014). These traps act passively to measure both abundance and activity levels of invertebrates, and as such, have been used as a proxy for invertebrate availability for insectivorous birds in the Arctic (Bolduc et al., 2013; Schekkerman et al., 1998, 2003). In 2010–2013, traps were placed near one of the six plots, with five traps spaced 15 m apart along one transect in mesic habitat and a similar arrangement in xeric habitat. To validate that invertebrate abundance patterns were similar across our plots, we changed this arrangement in 2014–2016 and instead placed four traps (two in mesic and two in xeric tundra) near each of four plots spread throughout our study area. Subsequent analyses of these 2014–2016 data showed that invertebrate biomass was correlated (*r* = 0.51–0.93/year) and of similar magnitude across these widely spaced plots, indicating that our sampling near a single plot in 2010–2013 was reflective of the entire study area. We typically sampled traps every 3 days between early June and late July, and restricted analyses to traps sampled on the same day after the date of 20% snow cover. Individual prey items were identified to family or order and length was measured to the nearest 0.25 mm for individuals <2 mm and to the nearest 0.5 mm for individuals >2 mm. We calculated mass for each individual using published length‐mass regression equations based on taxon (Ganihar, 1997; Gowing & Recher, 1984; Hawkins, Lankester, Lautenschlager, & Bell, 1997; Hódar, 1996; Lang, Kroob, & Stumpf, 1997; Rogers, Buschbom, & Watson, 1977; Sabo, Bastow, & Power, 2002; Sage, 1982; Sample, Cooper, Greer, & Whitmore, 1993; Schoener, 1980; Wrubleski & Rosenberg, 1990).

We estimated total biomass per trap day by combining the biomass of adult Diptera, Coleoptera, and Araneae. These taxa comprised the majority of items in the diet of shorebird chicks in this region (Holmes, 1966; Holmes & Pitelka, 1968). We did not consider insect larvae as they were reported to be unimportant to chicks less than two weeks old (Holmes, 1966; Holmes & Pitelka, 1968). We also removed large‐bodied invertebrates (i.e., >5 mg dry mass; accounting for 4%–9% of the total biomass in any given year) prior to biomass calculations because shorebird chicks were incapable of eating such large prey (i.e., they are gape‐limited; Pearce‐Higgins & Yalden, 2004, Schekkerman & Boele, 2009; D. Gerik, pers. comm.). As invertebrate biomass per trap day was highly correlated (*r* = 0.71–0.92/year) between habitat types (i.e., mesic or xeric), we combined information across habitats in all analyses.

#### 2.2.3 Shorebird hatch dates

We located shorebird nests using single‐person area searches, two‐person rope drags, and opportunistically (see Saalfeld & Lanctot, 2015 for detailed methods and effort). We visited nests found with fewer than four eggs (modal clutch size for all species) until clutches were completed, or until clutch size remained unchanged for two consecutive days. We estimated nest initiation dates (i.e., date first egg laid) assuming one egg was laid per day, and for nests found during incubation using egg flotation to estimate the start of incubation (i.e., date 4th egg was laid; Liebezeit et al., 2007). We checked nests every 3–5 days until 3–4 days prior to the estimated hatch date; at which time we checked nests every 2 days until eggs were starred (i.e., hatching was initiated), and daily thereafter. We defined a nest as successful when at least one egg hatched (Mayfield, 1975). See Saalfeld and Lanctot (2015) for evidence used to determine hatching or failure. If evidence at the nest was not conclusive, we classified the nest fate as unknown. For all analyses, we used actual hatch dates for successful nests and estimated hatch dates for unsuccessful and unknown fate nests. We excluded all nests in which hatch date was not estimated (e.g., nest depredated prior to floating eggs).

#### 2.2.4 Chick growth rates

We obtained growth rate data for known‐aged Dunlin (*Calidris alpina,* 2013, 2014, 2016), Pectoral Sandpiper (*C. melanotos*, 2013–2016), and Red Phalarope (*Phalaropus fulicarius,* 2013–2016) chicks. We captured chicks at hatch, weighed them to the nearest 0.1 g with an electronic scale, and marked them with a U.S. Geological Survey metal leg band. To relocate chicks, we attached a radio transmitter to one chick (Model A2414 weighing 0.3 g; Advanced Telemetry Systems [ATS], Isanti, Minnesota or Model LB‐2X weighing 0.26 g; Holohil Systems, Ltd) and one attending adult (i.e., male for Dunlin and Red Phalarope; female for Pectoral Sandpiper; Model A2415 or A2435 weighing 0.5–0.75 g; ATS) per brood. Transmitters were glued on the back of adults and chicks approximately 1 cm above the uropygial gland after feather clipping (Warnock & Warnock, 1993). We attempted to relocate and weigh chicks every 3 days. Additionally, we opportunistically recaptured and weighed banded chicks from other broods as encountered. We found that the attachment of the radio transmitter had little impact on chick growth, as chicks with transmitters weighed, on average, only 0.11 g less than the average weight of their other brood members at the time of last recapture (*n* = 34 broods; 11 Dunlin, 9 Pectoral Sandpiper, 14 Red Phalarope).

### 2.3 Data analyses

#### 2.3.1 Invertebrate availability

To determine how inter‐ and intra‐annual changes in weather conditions influenced invertebrate availability, we modeled invertebrate biomass in relation to timing of snowmelt, daily weather variables (i.e., temperature, precipitation, and wind speed), and growing degree days (GDD) using a general linear mixed model with year as a random effect (PROC MIXED, SAS Institute, Inc.). More specifically, our response variable was invertebrate biomass (calculated as total biomass per trap day) estimated for each invertebrate sampling period (i.e., period between invertebrate trap checks, typically 3 days). For predictor variables, we included the annual date of 20% snow cover calculated as the mean estimate across all plots for a given year. To account for weather‐related daily activity patterns of invertebrates, we included daily estimates for temperature and wind speed (i.e., hourly temperature and wind speed averaged across a 24‐hr period; data obtained from the National Climate Data Center; www.ncdc.noaa.gov; accessed 1 February 2017; station ID# 27502 located at the Wiley Post–Will Rogers Memorial Airport ~ 5–10 km from our study plots) averaged across each invertebrate sampling period, as well as the percentage of days any precipitation fell (including days with unmeasurable “trace” amounts) during each invertebrate sampling period. Finally, we calculated GDD by summing positive average daily temperatures (i.e., >0°C) since the date of 20% snow cover up to and including the end of each invertebrate sampling period. We included GDD as a quadratic term to account for insect emergence and depletion throughout the season. Prior to analyses, we standardized all fixed effects to have a mean of 0 and standard deviation of 1. We created additive models by combining nonhighly correlated (*r* < 0.6) environmental variables. In this and all subsequent analyses involving multiple models, we considered the model with the lowest AIC_c_ (Akaike's Information Criterion corrected for sample size) value to be the best‐fitting and models with a ΔAIC_c_ < 2 to be plausible (Burnham & Anderson, 2002).

#### 2.3.2 Estimates of phenological mismatch and relation to snowmelt

We estimated the degree of phenological mismatch by calculating the number of days between peak insect emergence and peak shorebird hatch. For each year, we defined peak insect emergence using the maximum value (i.e., vertex) of the quadratic function for GDD in the top‐ranked model predicting invertebrate abundance (see “Invertebrate availability” section in the Methods and Results), while peak shorebird hatch was defined as the median hatch date for all species combined (or for a given species; see Figure S1). We then linearly regressed the degree of phenological mismatch against the average date of 20% snow cover across all study plots in a given year (PROC REG, SAS Institute, Inc.).

#### 2.3.3 Impact of phenological mismatch on food availability

To determine the relationship between the degree of phenological mismatch and the amount of food available to chicks, we first estimated the amount of invertebrate biomass available (or expected to be available for unsuccessful and unknown fate nests) to chicks that were 2–10 days old by averaging daily estimates (assuming invertebrate biomass was the same for each day within each of our 3‐day sampling periods) over the 9‐day period for each brood. We excluded the first day after hatch because chicks rely on their yolk sac for the first day after hatching (Nice, 1962; Norton, 1973) and do not grow during this time (see “Impact of phenological mismatch on chick growth” section below). We focused on the first ten days after hatch because this time period is thought to correspond to peak energetic demands of chicks. For instance, Arctic‐breeding shorebird chicks obtain 25% of adult body mass between 3 and 9 days of age (dependent on species; Kwon et al., 2019)—a time when their basal metabolic rate is thought to peak (Ricklefs, 1973). This is also the time period when chicks are brooded during inclement weather, reducing foraging time (Krijgsveld, Reneerkens, McNett, & Ricklefs, 2003). After determination of the average invertebrate biomass available to broods 2–10 days old, we then linearly regressed these values for all species combined (or for a given species; see Figure S2) against the degree of phenological mismatch (PROC REG, SAS Institute, Inc.).

#### 2.3.4 Impact of phenological mismatch on chick growth

To determine the relationship between the degree of phenological mismatch and chick growth, we first generated growth curves for Dunlin, Pectoral Sandpiper, and Red Phalarope chicks for their first 18–20 days using mass from known‐age individuals and two growth models (i.e., Gompertz and logistic; PROC NLMIXED, SAS Institute, Inc.). The Gompertz growth model calculates age‐specific mass (*M*) in grams by: *M* = *A*·exp(−exp(−*K*·(*t*−*i*))) while the logistic model calculates *M* by: *M* = *A*/(1 + exp(−*K*·(*t*−*i*))), where *A* = asymptotic body mass of adults in grams, *K* = growth coefficient, *t* = age of the chick in days, and *i* = age at the point of inflection (Starck & Ricklefs, 1998). To control for repeated measurements on the same individuals, we included individual as a random effect in all models. We then compared both models using AIC_c_ values to determine the best‐fitting model for each species.

Next, we determined the relative importance of timing of hatch, weather, and food availability in explaining variation in chick growth using linear mixed‐effects models (PROC MIXED, SAS Institute, Inc). Here, our response variable was a chick's residual mass (observed—expected mass derived from the best‐fitting growth model) divided by its mass at each recapture event in which chicks were >1 day old (hereafter referred to as chick growth index). Fixed effects included five covariates: seasonal hatch date, temperature, percentage of days with precipitation, wind speed, and invertebrate biomass. Seasonal hatch date was defined as the number of days a nest hatched after the annual date of 20% snow cover (i.e., mean estimate across all plots for a given year). We calculated temperature, wind speed, and invertebrate biomass for each recapture event by averaging daily values (as defined above) from the date of hatch to the day before recapture. We also calculated the percentage of days with precipitation from the date of hatch to the day before recapture. Prior to analyses, we standardized all fixed effects to have a mean of 0 and standard deviation of 1. Due to nonlinear relationships, we transformed invertebrate biomass using a negative reciprocal transformation when this covariate was included in models for Dunlin and Red Phalarope. For Pectoral Sandpiper, however, models performed better (i.e., lower AIC_c_) when invertebrate biomass was included as a linear effect. We created additive and interaction models by combining nonhighly correlated (*r* < 0.6) variables; these models were restricted to ≤2 environmental covariates to correspond with our sample sizes. To account for nonindependence among measurements, we included year and individual nested within brood as random effects in all models.

For species in which the top‐ranked model included invertebrate biomass, we then calculated the percentage of broods that had sufficient food for average growth during the time chicks were 2–10 days old in each year. Here, the amount of food needed for average growth was estimated for each brood using the top‐ranked model coefficients to determine the value of invertebrate biomass at which the chick growth index was 0 (i.e., chick was growing at the rate predicted from the best‐fitting growth curve). For 5% of the nests, we could not determine whether sufficient food was available because there were <10 days of posthatch invertebrate data collected. For each species, we then linearly regressed the percentage of broods with sufficient food for average growth against the degree of phenological mismatch to determine how being mismatched with peak insect emergence affected chick growth (PROC REG, SAS Institute, Inc.).

#### 2.3.5 Timing of hatch in relation to food availability

To determine how timing of hatch influenced food availability, we investigated the influence of seasonal hatch date and date of 20% snow cover on the amount of invertebrate biomass available for chicks that were 2–10 days old using general linear mixed‐effects models with plot as a random effect. Here, our response variable was the average daily invertebrate biomass available to each brood that was 2–10 days old (see “Impact of phenological mismatch on food availability” section above), while the explanatory variables were seasonal hatch date and date of 20% snow cover for the plot in which the brood hatched. We included quadratic terms for both explanatory variables to investigate nonlinear trends, as well as their interactions. However, we did not include highly correlated (*r* > 0.6) variables in the same model.

## 3 RESULTS

### 3.1 Invertebrate availability

**Figure 1:**
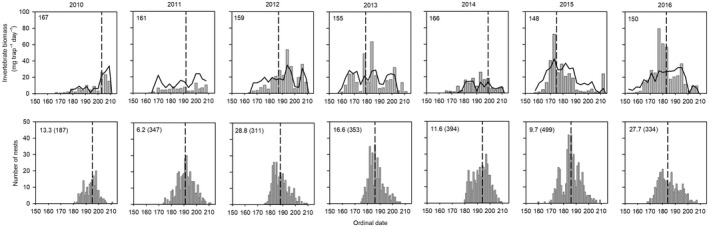
Actual (bars) and predicted (lines) invertebrate biomass (mg trap^−1^ day^−1^; top row) and the number of shorebird nests hatching or predicted to hatch (all species combined, bottom row) in relation to date near Utqiaġvik, Alaska, 2010–2016. Dashed vertical lines in top graphs correspond to the date when 107°C growing degree days was achieved each year (i.e., the predicted peak insect emergence date from the top‐ranked model; Table 1), while dashed vertical lines in the bottom graphs correspond to the median (i.e., peak) hatch date for all shorebird species within each year. Ordinal dates in the upper left of the top graphs correspond to the average date of 20% snow cover across all study plots. Values in the upper left of the bottom graphs correspond to the average invertebrate biomass available to broods 2–10 days old (mg trap^−1^ day^−1^; sample sizes in parentheses). Ordinal date 150 = 30 May (29 May in leap years). Seasonal variation in invertebrate biomass for major orders and the most abundant families within orders are in Figure S1

From 2010 to 2016, we captured and identified >200,000 individual invertebrates over 397 trap days. Invertebrate biomass and availability varied substantially within and among years (Figure 1). Total invertebrate biomass was dominated by the order Diptera, which was often >10 times the biomass of the other two orders, Araneae and Coleoptera, combined (Figure S1). Each invertebrate order, as well as families within orders, had their own, and often very different patterns of availability (Figure S1). The order Araneae occurred in low numbers throughout the season each year, whereas within the orders Diptera and Coleoptera, the most abundant families had very different seasonal peaks in availability across years (Figure S1).

The top‐ranked model predicting invertebrate biomass included GDD, temperature, percentage of days with precipitation, wind speed, and date of 20% snow cover (Table 1). Based on this model, insect emergence followed a quadratic relationship, peaking when GDD reached 107°C; with greater invertebrate biomass occurring in early snowmelt years (Table 2; Figure 1). Departures from this simple quadratic relationship occurred, however, due to daily weather patterns influencing invertebrate activity. Specifically, greater temperatures and lower wind speeds resulted in greater invertebrate activity (Table 2, Figure 1). The percentage of days with precipitation was also included in the top‐ranked model, however, the 95% confidence interval included zero, suggesting it was an uninformative parameter (Arnold, 2010).

**Table 1:** Model selection results predicting invertebrate biomass in relation to growing degree days (GDD; included as a quadratic term), temperature (temp), percentage of days with precipitation (precip%), wind speed (wind), and date of 20% snow cover (snow) near Utqiaġvik, Alaska, 2010–2016

Model^a^	*K* b	ΔAIC_c_ c	*w_i_* d
GDD^2^ + temp + precip% + wind + snow	7	0.0	0.79
GDD^2^ + temp + wind + snow	6	3.2	0.16
GDD^2^ + temp + precip% + wind	6	6.9	0.03
GDD^2^ + temp + precip% + snow	6	8.5	0.01
GDD^2^ + temp + wind	5	10.5	0.00
Intercept	1	92.7	0.00

a Only the top five models and intercept‐only model are shown.

b Number of parameters.

c Difference between model's Akaike's Information Criterion corrected for sample size (AIC_c_) and the lowest AIC_c_ value.

d AIC_c_ relative weight attributed to model.

**Table 2:** Parameter estimates, standard errors, and 95% confidence intervals from the top‐ranked linear mixed‐effects model predicting invertebrate biomass in relation to growing degree days (GDD; included as a quadratic term), temperature (temp), percentage of days with precipitation (precip%), wind speed (wind), and date of 20% snow cover (snow) near Utqiaġvik, Alaska, 2010–2016

Parameter	Estimate	*SE*	95% CI	
Intercept	17.924	1.995	14.015	21.833
GDD	−0.620	1.543	−3.645	2.405
GDD^2^	−4.095	1.185	−6.419	−1.772
Temp	7.506	1.355	4.851	10.161
Wind	−2.816	1.097	−4.966	−0.666
Precip%	−1.191	1.053	−3.255	0.873
Snow	−3.738	1.685	−7.040	−0.436

### 3.2 Estimates of phenological mismatch and relation to snowmelt

**Figure 2:**
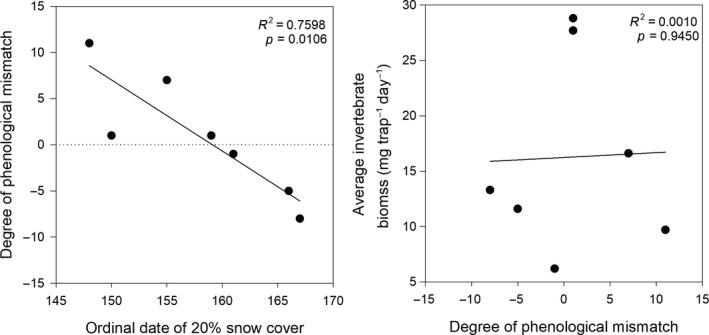
Degree of phenological mismatch (i.e., number of days between peak insect emergence and peak shorebird hatch) for all species combined relative to timing of snowmelt (left graph) and average invertebrate biomass available to broods (mg trap^−1^ day^−1^) that were 2–10 days old (right graph) near Utqiaġvik, Alaska, 2010–2016. The dashed line in the left graph indicates when peak shorebird hatch and insect emergence occurred at the same time; values above the line (positive values) indicate peak shorebird hatch occurred after peak insect emergence, while values below the line (negative values) indicate peak shorebird hatch occurred before peak insect emergence. Each point represents a year. Ordinal date 145 = 25 May (24 May in leap years)

Estimated peak insect emergence showed considerable variation among years, ranging from 24 June to 22 July, while peak shorebird hatch was less variable ranging from 2 to 14 July (Figure 1). When comparing timing between peaks, peak shorebird hatch occurred anywhere from 8 days before to 11 days after peak insect emergence (median = 1 day after peak insect emergence; *n* = 7). In general, the number of days between peak insect emergence and shorebird hatch was negatively related to timing of snowmelt (*F*_1,5_ = 15.81; *β* = −0.985), so that shorebirds tended to hatch after peak insect emergence in early snowmelt years, but before peak insect emergence in late snowmelt years (Figure 2). Similar trends were also noted within individual species (Figure S2).

### 3.3 Impact of phenological mismatch on food availability

We found that the average invertebrate biomass available to broods that were 2–10 days old was highly variable among years ranging from 6.2 to 28.8 mg trap^−1^ day^−1^ (*n* = 162–495 nests per year, Figure 1), but was unrelated to the degree of phenological mismatch (*p* > 0.05; Figure 2). Similar trends were also noted within individual species, with all species having similar estimates of average invertebrate biomass available to broods when averaged across years (13–17 mg trap^−1^ day^−1^; Figure S2).

### 3.4 Impact of phenological mismatch on chick growth

**Figure 3:**
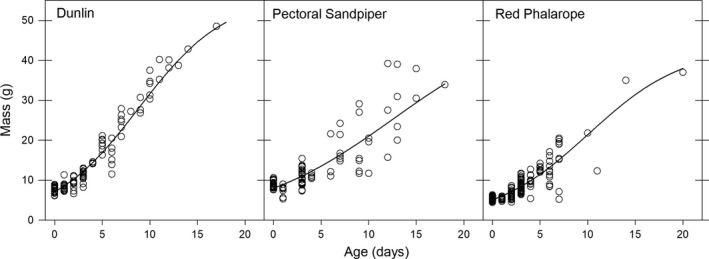
Observed (points) and predicted (lines) mass from logistic growth models predicting chick mass in relation to age in three shorebird species near Utqiaġvik, Alaska, 2013–2016

We obtained 118 mass measurements from Dunlin chicks (70 recaptures of 49 chicks from 23 broods; individuals captured 2–7 times), 116 mass measurements from Pectoral Sandpiper chicks (71 recaptures of 45 chicks from 23 broods; individuals captured 2–6 times), and 243 mass measurements from Red Phalarope chicks (131 recaptures of 115 chicks from 44 broods; individuals captured 2–3 times) when they were between 0 and 20 days of age. The logistic growth curve (Figure 3) was the best‐fitting model to describe variation in chick mass by age for all species (AIC_c_ = 451.3 vs. 470.1 for Gompertz growth curve in Dunlin, 555.5 vs. 558.2 for Pectoral Sandpiper, 844.1 vs. 852.7 for Red Phalarope).

**Figure 4:**
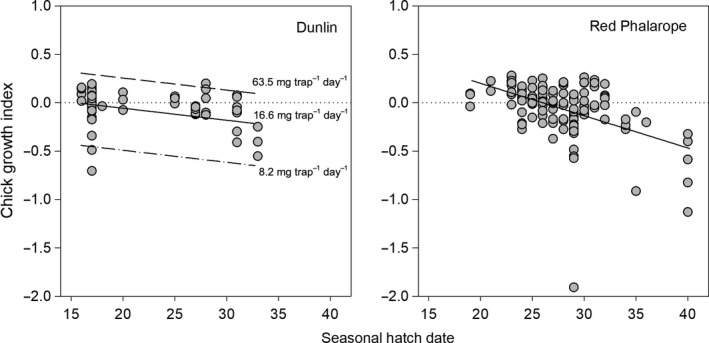
Dunlin and Red Phalarope chick growth indices (see text for definition) relative to seasonal hatch date (i.e., number of days a nest hatched after the date of 20% snow cover) from top‐ranked linear mixed‐effects models (see Table 3) near Utqiaġvik, Alaska, 2013–2016. For Dunlin, we illustrate the effect of invertebrate biomass (present as an additive effect in the top‐ranked model along with seasonal hatch date) at three levels representing the minimum (dot‐dashes), average (solid), and maximum (dashes) values during the 2–10 day posthatch period. The horizontal dotted line in each graph indicates average growth

**Figure 5:**
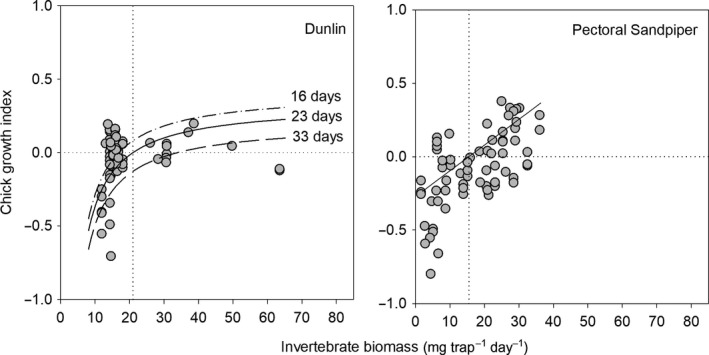
Dunlin and Pectoral Sandpiper chick growth indices (see text for definition) relative to invertebrate biomass from top‐ranked linear mixed‐effects models (see Table 3) near Utqiaġvik, Alaska, 2013–2016. For Dunlin, we illustrate the effect of seasonal hatch date (present as an additive effect in the top‐ranked model along with invertebrate biomass) at three levels representing the minimum (dot‐dashed), average (solid), and maximum (dashed) values during the 2–10 day posthatch period. The horizontal dotted line in each graph indicates average growth; vertical dotted line represents the amount food needed for average chick growth (see text)

We found that seasonal hatch date and food availability were the most influential factors on chick growth indices, although the importance of these variables differed among species (Table 3). Specifically, we found that chick growth indices were negatively related to seasonal hatch date in Dunlin (*β* = −0.07 ± 0.02) and Red Phalarope (*β* = −0.14 ± 0.03; Figure 4), and positively related to invertebrate biomass in Dunlin (*β* = 0.14 ± 0.03) and Pectoral Sandpiper (*β* = 0.18 ± 0.02; Figure 5). Further, we found that average growth for Dunlin and Pectoral Sandpiper chicks occurred when invertebrate biomass was 21 and 15.5 mg trap^−1^ day^−1^, respectively (estimate for Dunlin based on average hatch date; see vertical dotted lines on Figure 5).

**Table 3:** Linear mixed‐effects models predicting shorebird chick growth indices (see text for definition) in relation to seasonal hatch date, temperature (temp), percentage of days with precipitation (precip%), wind speed (wind), and invertebrate biomass (invert biomass) near Utqiaġvik, Alaska, 2013–2016

Model^a^	*K* b	ΔAIC_c_ c	*w_i_* d
*Dunlin*			
Seasonal hatch date + invert biomass	3	0.0	0.65
Seasonal hatch date*invert biomass	4	2.8	0.16
Invert biomass	2	4.8	0.06
Invert biomass + precip%	3	5.6	0.04
Temp*seasonal hatch date	4	6.5	0.03
Intercept	1	13.4	0.00
*Pectoral Sandpiper*			
Invert biomass	2	0.0	0.77
Seasonal hatch date	2	3.9	0.11
Temp + invert biomass	3	5.6	0.05
Temp*invert biomass	4	6.5	0.03
Wind*seasonal hatch date	4	7.4	0.02
Intercept	1	28.6	0.00
*Red Phalarope*			
Seasonal hatch date	2	0.0	0.73
Seasonal hatch date + temp	3	4.5	0.08
Seasonal hatch date + invert biomass	3	4.9	0.06
Seasonal hatch date + wind	3	5.4	0.05
Seasonal hatch date + precip%	3	5.5	0.05
Intercept	1	19.4	0.00

a Only the top five models and intercept‐only model are shown.

b Number of parameters.

c Difference between model's Akaike's Information Criterion corrected for sample size (AIC_c_) and the lowest AIC_c_ value.

d AIC_c_ relative weight attributed to model.

**Figure 6:**
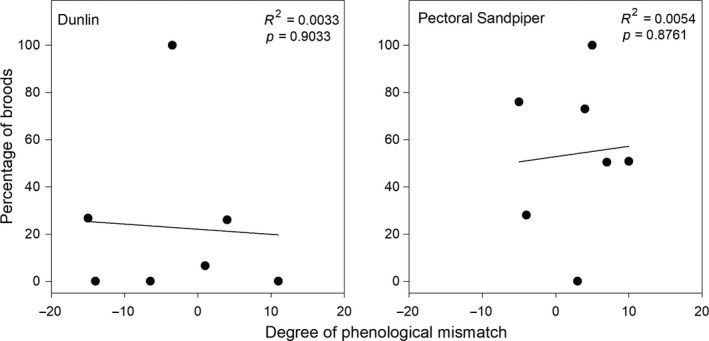
Percentage of Dunlin and Pectoral Sandpiper broods that had sufficient food for average growth (estimated from the top‐ranked models predicting chick growth indices; see Table 3) when chicks were 2–10 days old in relation to the degree of phenological mismatch (i.e., number of days between peak insect emergence and shorebird hatch) near Utqiaġvik, Alaska, 2010–2016

For Dunlin and Pectoral Sandpipers, where our top‐ranked model predicting chick growth indices included food availability, we found that the percentage of broods 2–10 days old that had sufficient food for average growth was highly variable among years, ranging from 0% to 100% in both species, but was unrelated to the degree of phenological mismatch (Figure 6). Averaging across years, we found fewer Dunlin broods (22.8 ± 36.1%) had sufficient food for average growth as compared to Pectoral Sandpipers (54.1 ± 33.1%). It should be noted, however, that both average invertebrate biomass and percentage of broods with sufficient food for average growth would likely have been lower had we had invertebrate data for the late‐hatching broods (5% of total).

### 3.5 Timing of hatch in relation to food availability

**Figure 7:**
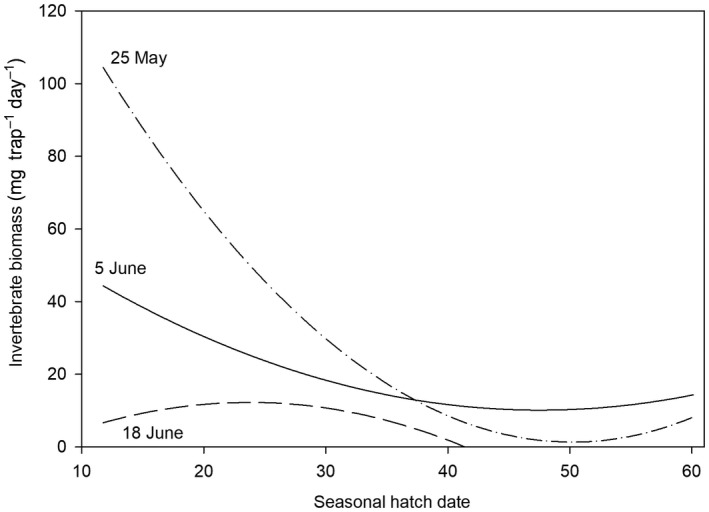
Predicted invertebrate biomass (mg trap^−1^ day^−1^) available to shorebird broods that were 2–10 days old relative to seasonal hatch date (i.e., number of days after 20% snow cover that a nest hatched) and date of 20% snow cover (illustrated at three levels representing early [dot‐dashes], average [solid], and late [dashes] snowmelt conditions) from the top‐ranked linear mixed‐effects model (see Table 4) near Utqiaġvik, Alaska, 2010–2016

For all species, we found that the best predictor of invertebrate biomass available to broods that were 2–10 days old was the interaction between the quadratic terms for seasonal hatch date and the date of 20% snow cover (Table 4). Broods from earlier‐laid nests generally had more invertebrate biomass available than later‐laid nests, especially in early snowmelt years (Figure 7). However, in both early and late snowmelt years, broods hatching late in the season (~ 40 days after the date of 20% snow cover) had very little food available to them (Figure 7).

**Table 4:** Linear mixed‐effects models predicting invertebrate biomass for shorebird broods that were 2–10 days old in relation to seasonal hatch date (number of days after the date of 20% snow cover a nest hatched) and date of 20% snow cover (snow) near Utqiaġvik, Alaska, 2010–2016

Model^a^	*K* b	ΔAIC_c_ c	*w_i_* d
Seasonal hatch date^2^*snow^2^	9	0.0	1.00
Seasonal hatch date^2^*snow	6	52.9	0.00
Seasonal hatch date*snow	4	200.6	0.00
Seasonal hatch date*snow^2^	6	202.2	0.00
Seasonal hatch date^2^ + snow^2^	5	375.1	0.00
Intercept	1	1,251.9	0.00

a Only the top five models and intercept‐only model are shown.

b Number of parameters.

c Difference between model's Akaike's Information Criterion corrected for sample size (AIC_c_) and the lowest AIC_c_ value.

d AIC_c_ relative weight attributed to model.

## 4 DISCUSSION

Our results indicate that Arctic‐breeding shorebirds have experienced increased phenological mismatch under earlier snowmelt conditions, with shorebirds tending to hatch after peak insect emergence in early snowmelt years, but before peak insect emergence in late snowmelt years. Previous studies have also noted high, but variable levels of phenological mismatch within shorebird species breeding throughout the Arctic (Kwon et al., 2019; McKinnon et al., 2012; Reneerkens et al., 2016; Senner et al., 2017). Although recent studies suggest shorebirds have some capacity to advance laying dates (Gill et al., 2014; Grabowski et al., 2013; Liebezeit et al., 2014; Saalfeld & Lanctot, 2017), advancement rates are likely limited by low plasticity in the start and progression of migration, which is controlled by a combination of endogenous and photoperiod cues (Karagicheva et al., 2016; Piersma et al., 2008). Therefore, advancing egg laying may be restricted to birds' ability to increase their speed of migration (Ely, McCaffery, & Gill, 2018; La Sorte & Fink, 2017) or to reduce the time between arrival and egg laying (Visser, Both, & Lambrechts, 2004). However, migration rates are limited by flight speeds, food availability at migration stop‐over sites, and weather conditions encountered during migration (La Sorte & Fink, 2017; Zhang et al., 2018). Similarly, reducing the time between arrival and egg laying may be difficult for shorebirds, as they are generally income breeders that must obtain food resources for egg development after arrival (Klaassen, Lindström, Meltofte, & Piersma, 2001; Morrison & Hobson, 2004). These facts are likely to prevent shorebirds from keeping pace with rising temperatures that are causing earlier snowmelt, thus precluding them from exploiting the progressively earlier availability of their invertebrate prey (Braegelman, 2016; Grabowski et al., 2013; Saalfeld & Lanctot, 2017).

While there is a very clear relationship between the degree of phenological mismatch and the timing of annual snowmelt, we failed to find any relationship between the degree of phenological mismatch and the amount of food available to chicks (Figure 2). This is likely the result of unpredictable weather conditions influencing the activity of invertebrates on the tundra surface, and thus, the ability of shorebird young to detect prey (Bolduc et al., 2013; Schekkerman et al., 1998, 2003; Tulp & Schekkerman, 2008). Even if shorebird chicks hatch during peak insect emergence, there is no guarantee they will be able to find sufficient food if invertebrate activity is low. We found that food available to 2–10 day old shorebird chicks was highly variable among years, and often inadequate for average growth. For example, average food ranged from 6.2 to 28.8 mg trap^−1^ day^−1^ across all species and years. For Dunlin and Pectoral Sandpiper, at least, only 20%–54% of broods had, on average across 7 years, sufficient food for average growth (Figure 6). We would expect similar estimates for the other six species in our study where no chick growth data were available, although larger species such as American Golden‐Plover and Long‐billed Dowitcher may require more food than the other, smaller species. Indeed, we found that only 36%–49% of broods of the other 6 species had sufficient food for average growth when assuming 15 mg trap^−1^ day^−1^ of invertebrate biomass was needed for average growth; these percentages decreased to 3%–24% when using 25 mg trap^−1^ day^−1^, which may be more realistic for larger species (Figure S3). Such results indicate that Arctic‐breeding shorebirds (at least currently, and potentially historically) experience highly variable levels of food availability even when hatching during peak insect emergence, potentially resulting in high annual variability in fledgling and first‐year survival rates. As a result, Arctic‐breeding shorebirds may be particularly vulnerable to any additional changes or stressors present away from the breeding grounds that decrease the ability of shorebirds to time their brood hatch with sufficient prey availability. It should be noted, however, that the average growth rates observed in this study were dependent upon the annual conditions experienced by the sampled chicks during our 4‐year study, and may be below growth rates that would have been obtained if environmental conditions were better, or food more plentiful (Loonstra, Verhoeven, & Piersma, 2018). This may explain why we observed lower growth rates and food requirements for Pectoral Sandpiper broods as compared to Dunlin, despite Pectoral Sandpipers reaching larger sizes in adulthood than Dunlin. The average growth rates documented here for Pectoral Sandpiper chicks may well be lower than what might be expected under ideal conditions. As such, the use of an average chick growth index as a benchmark to gauge whether broods have sufficient food may in fact be inadequate to determine chick fitness; additional data are needed to better understand how chick survival rates relate to food availability and seasonal weather patterns (see below).

Numerous researchers have postulated that shorebirds would benefit the most by hatching their young as early as possible (Meltofte et al., 2007; Schekkerman et al., 2003; Tulp & Schekkerman, 2008). Early nesting has been shown to maximize the probability of a brood hatching during peak invertebrate availability, enhancing the growth and survival of chicks (Loonstra et al., 2018; McKinnon et al., 2013, 2012; Pearce‐Higgins & Yalden, 2004; Reneerkens et al., 2016; Schekkerman et al., 2003; Senner et al., 2017). Our results reaffirm these benefits, as early hatch dates resulted in greater food availability and greater chick growth, especially in early to average snowmelt years (Figure 7). Early egg laying may also increase the chances for adults to re‐nest should their first nest fail (Gates, Lanctot, & Powell, 2013), and increase the time available for adults and chicks to acquire sufficient reserves prior to southbound migration, potentially allowing for earlier migrations (Meltofte et al., 2007; Taylor, Lanctot, Powell, Kendall, & Nigro, 2011; Tulp & Schekkerman, 2008).

The ability of shorebirds to nest early, however, is likely to depend on other selective pressures, such as seasonal variability in predation rates (Johansson, Kristensen, Nilsson, & Jonzén, 2015). For example, Reneerkens et al. (2016) suggested that greater predation on early‐hatching nests in Sanderling (*Calidris alba*) inhibited advancement of this species' nesting phenology. In contrast, Senner et al. (2017) suggested that greater predation on late‐hatching nests in Hudsonian Godwit (*Limosa haemastica*) selected for earlier nesting. Weiser et al. (2017) also documented a seasonal decline in daily nest survival in 8 of 22 Arctic‐breeding shorebirds at 16 sites spread across Russia, Alaska, and Canada. Similarly, a limited number of studies on brood survival indicate survival rates are often lower later in the nesting season (Hill, 2012; Ruthrauff & McCaffery, 2005). Thus, notwithstanding the Reneerkens et al. (2016) findings, greater survival rates of both early laid nests and early‐hatching chicks likely provide strong selection for Arctic‐breeding shorebirds to initiate nests as early as possible (although this is limited by the amount of plasticity in the start and progression of migration; see above).

Previous studies have suggested that warmer summer temperatures associated with climate change may provide physiological relief to shorebird chicks even though prey availability may decline (McKinnon et al., 2013). This is because warmer temperatures decrease energy expenditure of chicks needed for thermoregulation and the time chicks need to be brooded, increasing the amount of time available for chicks to forage (Krijgsveld et al., 2003; Schekkerman & Boele, 2009; Schekkerman et al., 2003). As a result, warmer temperatures can result in faster growth rates of shorebird chicks (McKinnon et al., 2013; Pearce‐Higgins & Yalden, 2002, 2004; Schekkerman et al., 1998, 2003; Senner et al., 2017). However, our results and others (Machín, Fernández‐Elipe, & Klaassen, 2018) suggest that daily weather is less important to shorebird chick growth than is invertebrate availability. Any positive effects warmer temperatures may provide could be negated by increased phenological mismatch between timing of shorebird hatch and invertebrate availability.

Additional studies are needed to better understand how chick survival rates relate to food availability and seasonal weather patterns. While we have assumed that poor chick growth indices lead to lower survival, it is unknown whether undernourished chicks can compensate for reduced food levels by growing more slowly over a longer period of time without compromising their survival. Additionally, we do not know how food availability relates to growth and survival of older chicks (i.e., >10 days old). Indeed, greater food requirements of older, larger chicks may make them even more vulnerable to food shortages. However, older chicks are more mobile and have additional foraging strategies (e.g., probing for insect larvae; Holmes, 1966, Holmes & Pitelka, 1968) that may allow them to access more food. Furthermore, little information is available concerning sex‐specific growth (especially in sexually dimorphic species) and how it relates to their susceptibility to changing environmental conditions. For example, Loonstra et al. (2018) documented that female Black‐tailed Godwits (*Limosa limosa*) grew faster than males during the prefledging period, suggesting a greater need for food, and thus, a greater vulnerability of females to poor environmental conditions.

Better data on shorebird diets will also improve our understanding of the potential implications of phenological mismatch on shorebird populations. While it is generally assumed that shorebird chicks consume all surface‐dwelling invertebrates, particular prey taxa and sizes are likely preferred, and some prey are potentially more nutritionally valuable (Twining et al., 2016). Additionally, prey consumed by various shorebird species may differ because of differences in how and where different shorebird species forage (e.g., preferred brood‐rearing habitat). In this study, we restricted the invertebrates used in our analyses to taxa and sizes previously documented as being consumed by shorebird young near Utqiaġvik (Holmes, 1966; Holmes & Pitelka, 1968; Pearce‐Higgins & Yalden, 2004). However, those data are based on a limited number of individuals from a few species where gut‐content analyses were conducted. Although these stomach analyses included Dunlin and Pectoral Sandpipers, no information was available for Red Phalarope chicks, which prefer more aquatic habitats than the other species. Thus, the importance of prey items may differ for Red Phalaropes and may not have been adequately sampled by our more terrestrial invertebrate traps. In addition, dietary analyses based on gut contents can have several drawbacks, including unequal digestion and retention of prey (Tollit, Wong, Winship, Rosen, & Trites, 2003), errors in identification of prey (Clare, Fraser, Braid, Fenton, & Herbert, 2009), and over‐simplification of prey composition due to difficult visual identification of closely related taxa. While new genetic techniques may improve our understanding of the prey items consumed (Gerik, 2018; Gerwing, Kim, Hamilton, Barbeau, & Addison, 2016; Novcic, Mizrahi, Veit, & Symondson, 2015; Symondson, 2002; Wirta et al., 2015), care must be used in implementing and interpreting these techniques as well (Oehm, Juen, Nagiller, Neuhauser, & Traugott, 2011; Valentini, Pompanon, & Taberlet, 2009). As different insect taxa have different emergence patterns (Butler, 1980, Høye & Forchhammer, 2008, Braegelman, 2016; and see Figure S1), dietary information is crucial to developing accurate prey availability curves (Vatka, Orell, & Rytkönen, 2016). Answers to these and other questions surrounding climate change effects on adult survival during the nonbreeding season are needed before we can determine the cumulative impacts of climate change on shorebird populations.

## CONFLICT OF INTEREST

None declared.

## AUTHOR'S CONTRIBUTIONS

STS, RBL, DCM, DCK, and MGB designed the study. STS, RBL, JAC, ACD, WBE, DEG, KG, PH, BLH, and BJL collected field data. STS and DCM performed statistical analyses. STS, DCM, and RBL wrote the paper with contributions from remaining authors.

## Data Availability

Data are available from the National Science Foundation's Arctic Data Center at https://doi.org/10.18739/A2VT1GP7Q.
