# Supplementary material for: Phenological mismatch in Arctic‐breeding shorebirds: Impact of snowmelt and unpredictable weather conditions on food availability and chick growth
Source: Ecol Evol. 2019 May 16;9(11):6693–707. doi: 10.1002/ece3.5248 (PMC6580279; doi:10.1002/ece3.5248)
Supplement: Supplementary file 1 [file ECE3-9-6693-s001.docx]

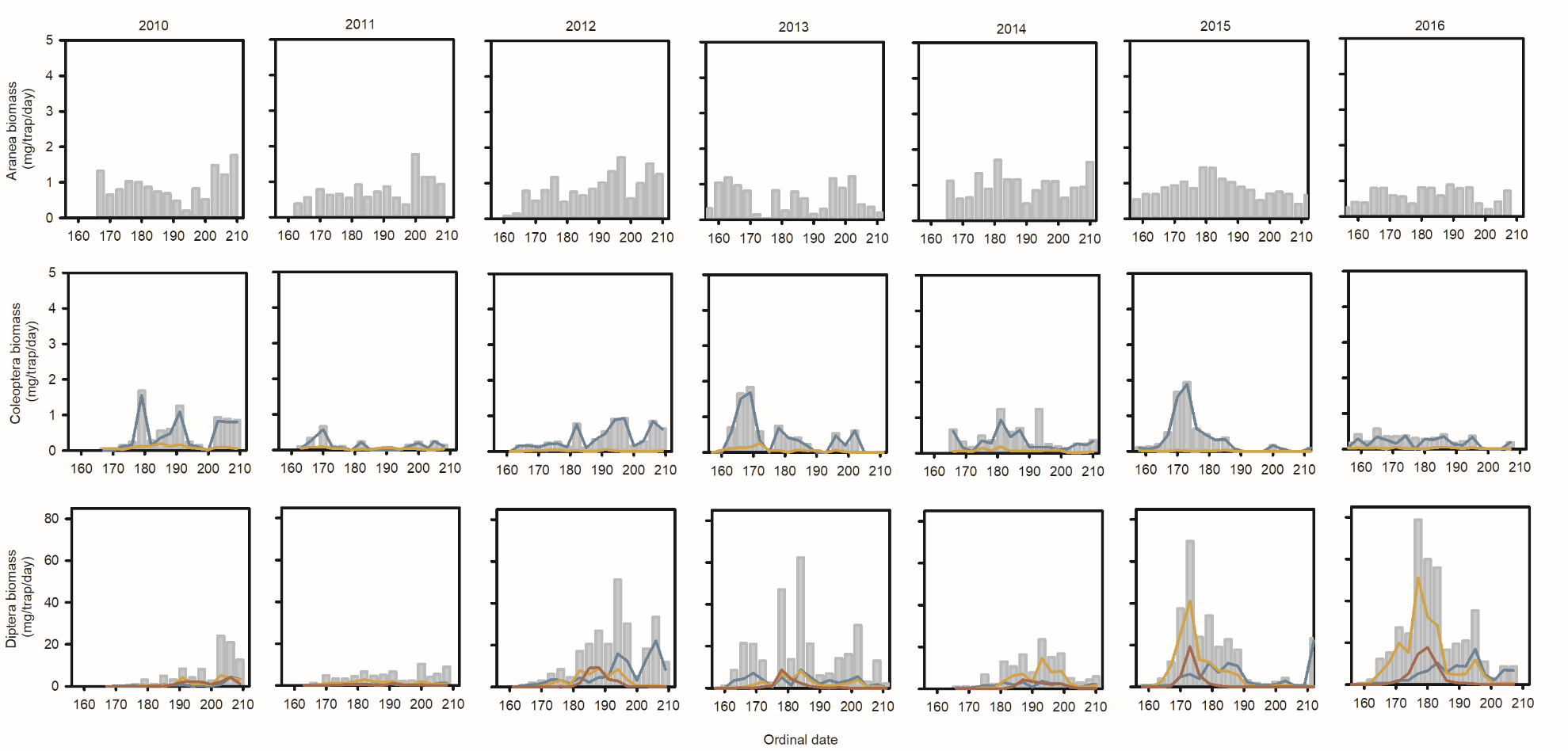


Figure S1. Seasonal variation in invertebrate biomass (mg/trap/day) for the orders Aranae (top row), Coleoptera (middle row, total shown in bars, Carabidae by blue line and Staphylinidae by yellow line), and Diptera (bottom row, total shown in bars, Brachycera by blue line, Chironomidae by yellow line, and Tipulidae by red line) near Utqiaġvik, Alaska, 2010–2016. Note difference in y-axis scale among the three invertebrate orders. Ordinal date 160 = 9 June (8 June in leap years).


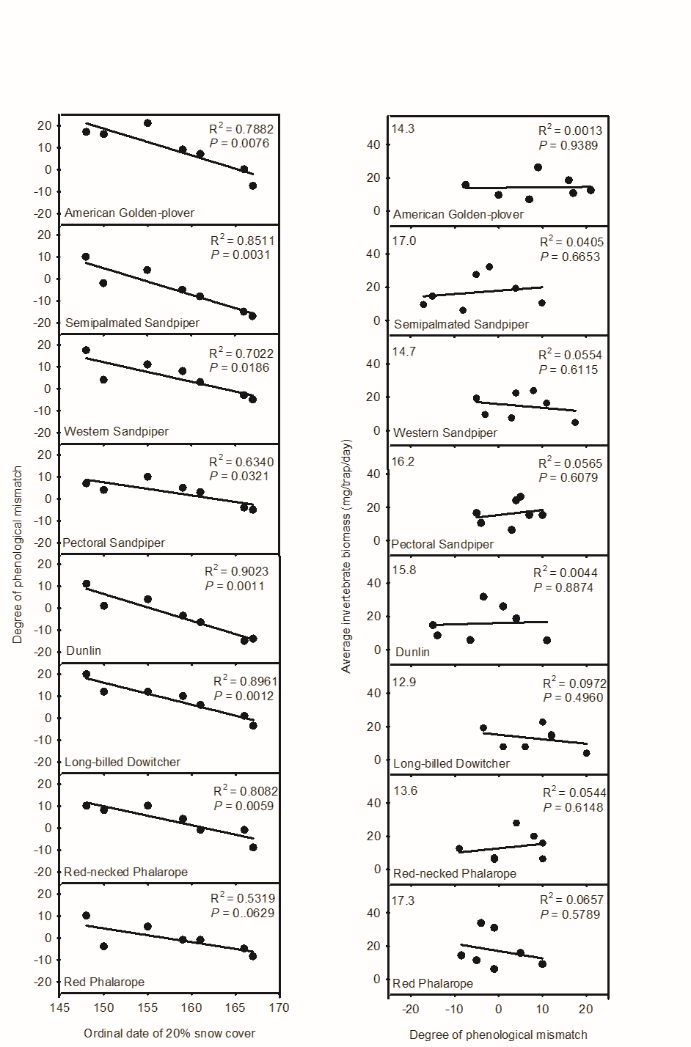


Figure S2. Degree of phenological mismatch (i.e., number of days between peak insect emergence and peak shorebird hatch) relative to timing of snowmelt (left graphs) and average invertebrate biomass available to broods (mg/trap/day) that were 2–10 days old (right graphs) for 8 species of shorebirds near Utqiaġvik, Alaska, 2010–2016. Each point represents a year. Values in the upper left of the right hand graphs correspond to the average invertebrate biomass available to broods 2–10 days old (mg/trap/day) averaged across years. Ordinal date 145 = 25 May (24 May in leap years).


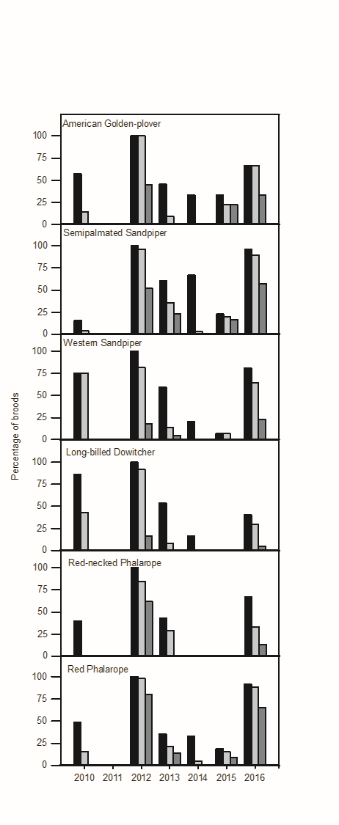


Figure S3. Annual estimates of the percentage of shorebird broods that had an average of 15mg/trap/day (black bars), 20 mg/trap/day (light grey bars), or 25 mg/trap/day (dark grey bars) of invertebrate biomass for 2–10 days after hatch near Utqiaġvik (formerly Barrow), Alaska, 2010–2016. No bars indicate a value of 0.
